# Supplementary material for: A cross-sectional survey of avian influenza knowledge among poultry farmworkers in Indonesia
Source: PeerJ. 2023 Jan 16;11:e14600. doi: 10.7717/peerj.14600 (PMC9851045; doi:10.7717/peerj.14600)
Supplement: Supplemental Information 3 [file peerj-11-14600-s003.docx]

**An Epidemiological Survey of Avian Influenza Knowledge and Practices among Poultry Farm Workers in Indonesia**

**Demographic variables**

1. Gender 1. Male 2. Female
2. Age <20-year 20-30-year 31-50 year
3. Residence 1.Urban, 2. Rural
4. Religion 1.Muslim, 2. Christian, 3. Hindu 4. Katolik
5. Educational Status 1. Non to primary 2. Higher than primary
6. Working Status 1. Farm owner 2. Paid employees

**Knowledge of respondents related to Avian Influenza**

**8.** Have you heard about avian influenza? 1) Yes 2) No

9. Source of Information about AI. 1. Radio, 2.TV, 3. Newspaper 4. Health workers 5.

Friends

10. Is Avian influenza is a contagious infection that affect all birds? 1) Yes 2) No 3)

Don’t know

**Mode of transmission**

11. Animal-to-animal 1) Yes 2) No 3) Don’t know

12. Animal-to-human 1) Yes 2) No 3) Don’t know

13. Human-to-human 1) Yes 2) No 3) Don’t know

14. Touching uncooked poultry 1) Yes 2) No 3) Don’t know

15.Touching uncooked eggs 1) Yes 2) No 3) Don’t know

**Vehicles of transmission**

1. Poultry 1) Yes 2) No 3) Don’t know
2. Birds 1) Yes 2) No 3) Don’t know
3. Other animals 1) Yes 2) No 3) Don’t know

**Risk groups**

1. Poultry workers 1) Yes 2) No 3) Don’t know
2. Butchers 1) Yes 2) No 3) Don’t know
3. Veterinarians 1) Yes 2) No 3) Don’t know

**Respondents’ practices related to avian Influenza**

22. Are you use of separate clothes? 1.Always 2. Sometimes 3. Never

23.Are you Contact with bird cages? 1.Always 2. Sometimes 3. Never

24.Use of face mask 1.Always 2. Sometimes 3. Never

25.Boots or boot covers 1.Always 2. Sometimes 3. Never

26.Handwashing with soap 1. Always 2. Sometimes 3. Never

27.Consult doctors 1.Always 2. Sometimes 3. Never

28.Dispose dead birds properly 1.Always 2. Sometimes 3. Never
