# Supplementary material for: A cross-sectional survey of avian influenza knowledge among poultry farmworkers in Indonesia
Source: PeerJ. 2023 Jan 16;11:e14600. doi: 10.7717/peerj.14600 (PMC9851045; doi:10.7717/peerj.14600)
Supplement: Supplemental Information 4 [file peerj-11-14600-s004.docx]

**Survei Epidemiologi Pengetahuan dan Praktik Flu Burung pada Buruh Peternakan Unggas di Indonesia**

Salam hormat untuk bapak/ibu sekalian. Perkenalkan saya Saifur Rehman, Mahasiswa S3 dari Fakultas Kedokteran Hewan Universitas Airlangga Surabaya. Mohon kepada bapak/ibu dapat membantu saya dengan mengisi kuisioner terkait flu burung. Terima kasih banyak.

**Variabel demografis**

1. Jenis Kelamin 1. Laki-laki, 2. Perempuan

2. Usia 1.<20 tahun 2. 20-30 tahun 3.31-50 tahun

3. Tempat Tinggal 1.Perkotaan, 2. Pedesaan

4. Agama 1.Muslim, 2. Kristen, 3. Hindu, 4. Katolik,

5. Status Pendidikan 1.Tidak Sekolah 2. Lebih tinggi dari SD

6. Status Kerja 1. Pemilik peternakan 2. Karyawan

**Pengetahuan responden Terkait Flu Burung**

8. Pernahkah Anda mendengar tentang flu burung? 1) Ya 2) Tidak

9. Sumber Informasi tentang flu burung? 1. Radio, 2.TV, 3. Surat Kabar 4. Tenaga Kesehatan 5. Teman-teman

10. Apakah Avian Influenza merupakan infeksi menular yang menyerang semua unggas?

1) Ya 2) Tidak 3) Tidak tahu

**Cara Penularan**

11. Hewan ke hewan 1) Ya 2) Tidak 3) Tidak tahu

12. Hewan-ke-manusia 1) Ya 2) Tidak 3) Tidak tahu

13. Manusia ke manusia 1) Ya 2) Tidak 3) Tidak tahu

14. Menyentuh unggas mentah 1) Ya 2) Tidak 3) Tidak tahu

15 Menyentuh telur mentah 1) Ya 2) Tidak 3) Tidak tahu

**Kendaraan Transmisi (penyebaran bawaan)**

16 Unggas Ya 2) Tidak 3) Tidak tahu

17 Burung Ya 2) Tidak 3) Tidak tahu

18 Hewan lain Ya 2) Tidak 3) Tidak tahu

**Kelompok risiko**

19 Pekerja unggas Ya 2) Tidak 3) Tidak tahu

20 Tukang Daging Ya 2) Tidak 3) Tidak tahu

21 Dokter Hewan Ya 2) Tidak 3) Tidak tahu

**Praktik responden Terkait flu burung**

22. Apakah Anda menggunakan pakaian terpisah? 1.Selalu 2. Kadang-kadang 3. Tidak

pernah

23.Apakah Anda Kontak dengan sangkar burung? 1.Selalu 2. Kadang-kadang 3. Tidak pernah

24.Penggunaan masker 1.Selalu 2. Kadang-kadang 3. Tidak pernah

25.Boots atau pent-up boot 1.Selalu 2. Kadang-kadang 3. Tidak pernah

26.Cuci tangan pakai sabun 1.Selalu 2. Kadang-kadang 3. Tidak Pernah

27.Konsultasikan dengan dokter 1.Selalu 2. Kadang-kadang 3. Tidak pernah

28.Membuang unggas mati dengan benar 1. Selalu 2. Kadang-kadang 3. Tidak pernah
